# Pretreatment of Mesenchymal Stem Cells with Electrical Stimulation as a Strategy to Improve Bone Tissue Engineering Outcomes

**DOI:** 10.3390/cells12172151

**Published:** 2023-08-26

**Authors:** Santiago Bianconi, Karla M. C. Oliveira, Kari-Leticia Klein, Jakob Wolf, Alexander Schaible, Katrin Schröder, John Barker, Ingo Marzi, Liudmila Leppik, Dirk Henrich

**Affiliations:** 1 Department of Trauma, Hand and Reconstructive Surgery, University Hospital, Goethe University Frankfurt, 60590 Frankfurt am Main, Germanykarileticia.klein@gmail.com (K.-L.K.); wolfdonner2@t-online.de (J.W.); alexanderschaiblex@gmail.com (A.S.); ingo.marzi@kgu.de (I.M.); liudmila.leppik@kgu.de (L.L.); d.henrich@trauma.uni-frankfurt.de (D.H.); 2 Vascular Research Centre, Goethe University Frankfurt, 60590 Frankfurt am Main, Germany; 3 Frankfurt Initiative for Regenerative Medicine, Experimental Orthopedics and Trauma Surgery, Goethe University Frankfurt, 60528 Frankfurt am Main, Germany; jhb121654@gmail.com

**Keywords:** electrical stimulation, bone healing, bone tissue engineering, critical size bone defect, bone marrow derived mesenchymal stem cells, beta-tricalcium phosphate

## Abstract

Electrical stimulation (EStim), whether used alone or in combination with bone tissue engineering (BTE) approaches, has been shown to promote bone healing. In our previous in vitro studies, mesenchymal stem cells (MSCs) were exposed to EStim and a sustained, long-lasting increase in osteogenic activity was observed. Based on these findings, we hypothesized that pretreating MSC with EStim, in 2D or 3D cultures, before using them to treat large bone defects would improve BTE treatments. Critical size femur defects were created in 120 Sprague–Dawley rats and treated with scaffold granules seeded with MSCs that were pre-exposed or not (control group) to EStim 1 h/day for 7 days in 2D (MSCs alone) or 3D culture (MSCs + scaffolds). Bone healing was assessed at 1, 4, and 8 weeks post-surgery. In all groups, the percentage of new bone increased, while fibrous tissue and CD68+ cell count decreased over time. However, these and other healing features, like mineral density, bending stiffness, the amount of new bone and cartilage, and the gene expression of osteogenic markers, did not significantly differ between groups. Based on these findings, it appears that the bone healing environment could counteract the long-term, pro-osteogenic effects of EStim seen in our in vitro studies. Thus, EStim seems to be more effective when administered directly and continuously at the defect site during bone healing, as indicated by our previous studies.

## 1. Introduction

Delayed and non-healing large bone defects caused by major trauma and the surgical removal of tumors or infected bone constitute major challenges for trauma, orthopedic, and maxillofacial surgeons. In these cases, reestablishing bone integrity is essential for restoring function. Several treatments are available, of which autologous bone grafts are considered to be the gold standard. The success of autologous grafts has been demonstrated to lie in their ability to provide bone-generating cells, signaling growth factors, and 3D structural bone material to refill the void left by missing bone. Nevertheless, autologous bone grafting and other conventional treatments are associated with complications and drawbacks, which continue to stimulate the search for alternative treatment approaches [1]. One such approach is bone tissue engineering, for which the outcomes from initial preclinical and clinical studies have been encouraging (Reviewed in [2,3]). Bone tissue engineering (BTE) treatments can mimic autologous bone grafts and have the added benefit of optimizing healing by allowing the manipulation of the “type” and/or “activity” of implanted cells, scaffolds, and growth factors. A great deal of effort has been/is being spent on optimizing different combinations of this mix and/or manipulations to improve outcomes in this exciting new treatment approach [4].

While the use of electrical stimulation (EStim) to treat bone fractures is not new, the understanding of the mechanisms by which EStim promotes bone healing has not yet been completely elucidated. Several recently published in vitro studies suggest that this pro-healing effect could be due to EStim’s effect on bone stem cell migration, proliferation, alignment, differentiation, and attachment to scaffold materials [5,6,7,8]. In addition, it has been shown that EStim increases mineralization, extracellular matrix deposition, and enhances the expression of several osteogenic genes [9,10]. It follows that, if methods were developed to control and regulate these cell behaviors and functions, they could be used to significantly improve BTE treatment outcomes.

In a previous in vivo study in a rat forelimb amputation model, we demonstrated that treating amputated limb stumps with EStim for 28 days resulted in significant new cartilage, bone, and vessel formation [11]. Based on these findings, we conducted in vitro experiments to examine the mechanisms by which EStim exerted this positive osteo-, chondro-, and angiogenic effect. In an EStim cell culture chamber, mesenchymal stem cells (MSCs) were exposed to different regimens of EStim, and it was observed that 100 mV/mm of direct current (DC) EStim for 1 h/day caused increased osteogenic differentiation, which was expressed in higher calcium deposition and gene expression of the osteogenic markers *RunX2*, *Osteopontin*, *Bmp2*, and *TGF-β_1_* [7,9].

In another in vitro study, our team seeded MSCs onto scaffold material to simulate a BTE treatment, EStim was applied using the abovementioned regime, and a significant increase in osteogenic differentiation was also observed in this 3D environment [12]. In another subsequent experiment, we found that the observed positive osteogenic effects persisted long after the discontinuation of EStim treatment [13], which is particularly relevant to the present work. This observation was confirmed using gene expression and alizarin red stain analysis, which showed that osteogenic markers and calcification levels remained elevated for 1 week after discontinuing EStim exposure.

The feasibility and effectiveness of combining EStim and BTE to treat large segmental bone defects was also assessed by our team. In a previous study, rat critical size femur defects were filled with beta-tricalcium phosphate (β-TCP) scaffold granules populated with MSCs and DC EStim (1 × 10^−6^–1 × 10^−7^ A) was applied directly to the defect using an implanted EStim device. The results showed a significant improvement in the rate and quality of bone healing after 8 weeks of EStim treatment [12]. However, despite this improvement in healing, surgically implanting, monitoring, and explanting the EStim devices proved to be a major challenge and led to multiple complications that would have been a major drawback in the future clinical application of this treatment approach.

The above-described surprising finding that pretreating MSCs with EStim in vitro resulted in a long-lasting positive osteogenic effect, combined with the challenges we encountered when delivering EStim to a bone defect with an implanted device in our in vivo model, led us to design the present experiment. The concept of pretreating stem cell–scaffold constructs with EStim prior to their use in BTE treatments is, to our knowledge, entirely new and has not been reported previously in the literature. In the current work, we assessed whether pretreating MSCs alone (2D) or on scaffolds (3D) with direct current EStim produces sustained long-lasting pro-osteogenic activity when used to treat large bone defects in a rat model. The rate and quality of bone healing were studied 1, 4, and 8 weeks post-surgery using micro-CT, bone strength, histological, and gene expression analysis.

## 2. Materials and Methods

### 2.1. Ethics and Animal Care

All animal experiments were performed according to the institutional guidelines and approved (Project Nr.: FK-1140) by our university’s animal care and oversight committee (Regierungspräsidium Darmstadt, Veterinärdezernat, Wilhelminenstraβe 1–3) according to German law. Animals were maintained on a 12:12 h light–dark regimen in an air-flow- and temperature-controlled room (21.8 °C). Pelleted food and water were provided ad libitum. A total of 159 nine-week-old female Sprague–Dawley (SD) rats (Charles River Labs Int., Sulzfeld, Germany) (270–300 g) were used in this study. A bone healing assessment was performed at 1-, 4-, and 8-week intervals after surgery. The animals were monitored daily for signs of pain, discomfort, and complications during the first postoperative week and subsequently twice a week for the duration of the experiment. 

### 2.2. Treatment Groups

**Figure 1:**
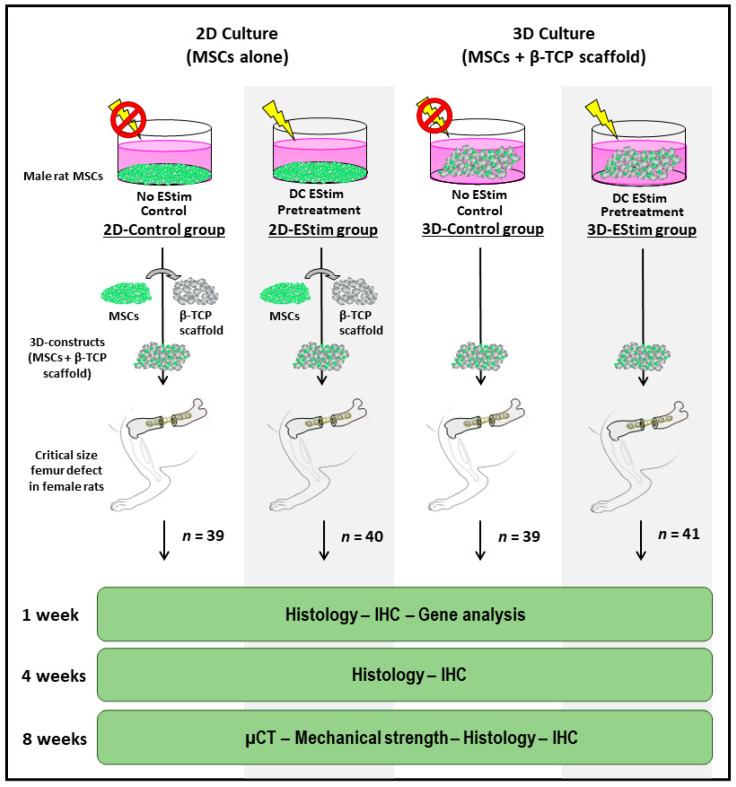
Group setup and procedures to assess bone healing at different post-surgical intervals. MSCs, rat bone marrow mesenchymal stem cells; DC EStim, direct current electrical stimulation; β-TCP, beta-tricalcium phosphate; IHC, immunohistochemistry, µCT, micro computed tomography; n, number of animals.

Female rats were operated to create a critical size femur defect and randomly allocated into four groups, each treated as follows: (1) MSCs cultured in 2D conditions non-treated with EStim and seeded onto β-TCP scaffolds  (2D-Control group; *n* = 39); (2) MSCs pretreated with EStim  in 2D conditions and seeded onto β-TCP scaffolds  (2D-EStim group; *n* = 40); (3) MSCs seeded onto β-TCP scaffolds and non-treated with EStim   (3D-Control group; *n* = 39); and (4) MSCs seeded onto β-TCP scaffolds and pretreated with EStim (3D-EStim group; *n* = 41) (Figure 1).

### 2.3. EStim Pretreatment of MSCs in 2D or 3D Conditions

**Figure 2:**
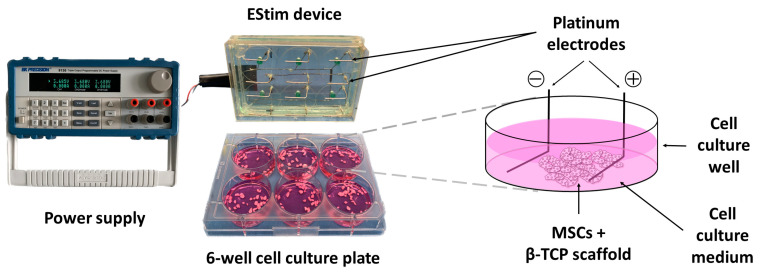
Electrical stimulation setup. Pretreatment of male rat mesenchymal stem cells (MSCs) seeded onto β-TCP scaffold granules (3D-EStim group) with direct current electrical stimulation (DC EStim) 100 mV/mm for 1 h/day during 7 days by means of a custom-made EStim cell culture device.

Sprague–Dawley rat MSCs from bone marrow (RASMX-01001) were purchased from Cyagen (Guangzhou, China). Frozen vials of cells were thawed and cultured as described in detail elsewhere [9]. MSCs (passage 6) were seeded alone (2D-groups) or in 3D constructs (3D-groups) with growth medium (GM, consisting of 10% fetal bovine serum (Gibco, Paisley, UK), 100 U/mL penicillin, and 100 µg/mL streptomycin (Sigma-Aldrich, St. Louis, MO, USA) in Dulbecco’s Modified Eagle Medium + GlutaMAX + 1 g/L D-glucose (Gibco, Paisley, UK)) in 6-well cell culture plates (TPP, Trasadingen, Switzerland) and placed in a humidified incubator at 37 °C, 5% O_2_, and 5% CO_2_. After 24 h of culture (day 1), GM was replaced with osteogenic differentiation medium (ODM, GM supplemented with 10^−6^ M dexamethasone (Sigma-Aldrich, St. Louis, MO, USA), 10 mM beta-glycerophosphate (Sigma-Aldrich, St. Louis, MO, USA), and 0.05 mM ascorbic acid (Stemcell, Cologne, Germany)). The cell density in the 2D-groups was 9 × 10^4^ cells/well. The 3D constructs consisted of 2 × 10^5^ cells seeded onto 90 mg β-TCP granules (ChronOS, DePuy Synthes, Oberdorf, Switzerland) (0.7–1.4 mm diameter, 60% porosity, 100–500 μm pore size) pre-soaked in medium. From day 2 to during the 1st week, cells from the 2D- and 3D-groups were exposed (experimental group) or not (control group) to 100 mV/mm DC EStim for 1 h/day using a custom-made EStim cell culture device (Figure 2) [7,12]. Culture medium was replaced every 3–4 days. On the day after the last EStim session (day 9), 2 × 10^5^ cells from the 2D-groups were seeded onto 90 mg β-TCP granules pre-saturated with medium and incubated for 1 h in a humidified atmosphere at 37 °C, 5% O_2_, and 5% CO_2_. Three-dimensional constructs from the 2D- and 3D-groups were used to treat critical size defects created on the female rat femora. In a separate experiment, MSCs were pretreated as described above, and the assessment of cell metabolic activity, seeding efficiency, and cell distribution on the scaffolds was performed on the constructs on the day after the last EStim session (day 9, immediately prior to their intended transfer into animals).

### 2.4. Seeding Efficiency

In order to determine the amount of MSCs attached to the β-TCP granules, seeding efficiency was assessed 24 h after seeding onto scaffold granules (on experimental day 1) in the 3D-groups and 1 h after seeding onto scaffold granules (on experimental day 9) in the 2D-groups. After transferring the 3D constructs to another plate, the remaining cells were treated with Accutase^®^ solution (Sigma-Aldrich, St. Louis, MO, USA), centrifuged, resuspended in PBS without Ca^2+^Mg^2+^ (Gibco, Paisley, UK), and manually counted with a hemocytometer (NanoEntek, Gyeonggi-do, Korea). The seeding efficiency was calculated as a percentage of recovered cells from the initially seeded amount (2 × 10^5^ cells).

### 2.5. Cell Metabolic Activity

The cell metabolic activity of MSCs on 3D constructs from the 2D- and 3D-groups was assessed using alamarBlue^®^ reagent (BIO-RAD, Feldkirchen, Germany) according to the manufacturer’s protocol. Briefly, after the aspiration of culture medium, the 3D constructs were washed twice with sterile PBS without Ca^2+^Mg^2+^ (Gibco, Paisley, UK). Then, 1 mL of fresh prewarmed medium with 100 µL of alamarBlue^®^ reagent was added to every well. The blank control was set in a well containing β-TCP granules. After 4 h of incubation at 37 °C, 5% CO_2_, and 5% O_2_, the absorbance of the cell and blank control samples was measured in triplicate at 570 and 600 nm using a plate reader (Tecan Infinite 200, Grodig, Austria). The absorbance values were used to calculate the percentage of resazurin reduction according to a formula described in the manufacturer’s protocol.

### 2.6. Cell Distribution on 3D Constructs

Cell distribution on scaffold granules was assessed via 4′,6-diamidino-2-phenylindole (DAPI) staining. The cells were treated as described in Section 2.3 and fixed with 4% paraformaldehyde (PFA) solution for 15 min at room temperature. After the removal of PFA, the cells were washed three times with PBS without Ca^2+^Mg^2+^ (Gibco, Paisley, UK) and stained with 1 mL of DAPI reagent (1:1000 in PBS) for 10 min at room temperature in the dark. Afterwards, staining solution was removed and the cells were washed four times with PBS and visualized with an Axioobserver Z1 fluorescence microscope (Zeiss, Gottingen, Germany). Images were captured with AxioVs40 version 4.7.2.0 software (Zeiss, Gottingen, Germany).

### 2.7. Rat Critical Size Femur Defect Creation

Under general anesthesia (Ketamine (Pfizer, Berlin, Germany), 100 mg/kg and xylazine hydrochloride (Bayer, Leberkusen, Germany), 10 mg/kg, IP), the right hind limbs of the rats were shaved, cleaned, and disinfected. The dermis and superficial fascia were incised 3 cm longitudinally over the femur. The tensor fascia lata, biceps femoris, and vastus lateralis muscles were elevated from the greater trochanter, exposing the lateral aspect of the femur. Two proximal and two distal 1.5 mm cortical screws (DePuy Synthes, Oberdorf, Switzerland) were used to fix a five-hole plate (DePuy Synthes, Oberdorf, Switzerland) to the lateral aspect of the femur. After securing the plate in place, a 0.22 mm gigli wire saw (RISystem, Landquart, Switzerland) was used to create a 5 mm long defect on the femur shaft beneath the mid-point of the plate. The bone defects were filled with 3D constructs according to their respective treatments and irrigated with sterile saline. The superficial fascia and the skin were reapproximated and sutured with 3–0 Vicryl and 4–0 Prolene (Ethicon, Norderstedt, Germany), respectively [12,14,15,16,17].

### 2.8. Bone Healing Assessment

At the corresponding healing time points, animals were euthanized via CO_2_ inhalation. Subsequently, their femora were dissected free and subjected to macro- and microscopic examination to detect signs of infection or tumors. Plates and screws were removed and the bones were snap frozen in liquid nitrogen and stored at −80 °C for subsequent gene expression analysis, or wrapped in gauze soaked in PBS without Ca^2+^Mg^2+^ and stored at −80 °C for later assessments. To reduce the number of animals, the same bones were first submitted to micro-CT analysis and mechanical testing before undergoing histological assessment [15,18,19]. To trace implanted cells within the tissues formed in the bone defect area, Y-chromosome in situ hybridization was conducted.

### 2.9. Micro-Computed Tomography Analysis

Micro-computed tomographic (µCT) analysis of the collected femora was performed by the Vascular Research Centre in Frankfurt am Main, Germany, using a high-resolution µCT Skyscan 1176 (Bruker AXS, Karlsruhe, Germany). Bones collected after 8 weeks of healing were thawed and stored in 70% ethanol until analysis. The femora were placed with the long axis orthogonally to the X-ray-beam (Al: 0.5 mm; voltage: 50 kV; current: 500 μA; frame average: 7; rotation ra.: 180; rotation st.: 0.5). Two-dimensional CT-images of each bone were scanned and then reconstructed using a standard back convolution procedure. The reconstructed images were saved in 3D arrays [15]. The ratio of new bone volume to total defect volume was determined using CT-Analyzer version 1.18 software (Bruker AXS, Karlsruhe, Germany) and ImageJ version 1.53g software (National Institutes of Health, Bethesda, MD, USA) [19,20].

### 2.10. Bone Mechanical Strength Assessment

After μCT scanning, the bones were stored in 70% ethanol at 4 °C until they were subjected to a destructive 3-point bending test with a universal material testing machine (Test machine Zwickiline 5.0, Zwick-Roell, Ulm, Germany), as described elsewhere [16]. In brief, femora were placed on two rounded bars spaced 20 mm apart, positioning the bone defect zone at the midpoint between the bars. The process of “bending until failure” was executed by gradually lowering one bar onto the femur, maintaining a consistent deflection rate of 0.1 mm/s. During this procedure, the load and deflection values were continuously recorded. Based on the collected data, a graph illustrating the relationship between load and displacement was created and maximal load, yield load, and stiffness were calculated with TestXpert-II version 3.3 software (Zwick-Roell, Ulm, Germany). Contralateral femora were used as control specimens, and the data are presented as a percentage of the ultimate load of the corresponding contralateral femur [21].

### 2.11. Histological Analysis

For a histological evaluation of the defect tissues, femora collected at the 1-, 4-, and 8-week post-surgery periods were fixed in Zinc-Formal-Fixx solution (Thermo Scientific, Kalamazoo, MI, USA) for a period of 24 h. Following this step, they underwent a 14-day decalcification process in 10% EDTA/TRIS-HCl (pH 7.4). Subsequently, the bones were embedded in paraffin for later histomorphometric analysis. Tissue sections measuring 5–7 µm in thickness were taken parallel to the femur’s long axis and stained with (1) Movat pentachrome staining (to assess bone healing), (2) immunohistochemistry for α-smooth muscle actin (α-SMA) (to visualize new vessel formation), and (3) immunohistochemistry for CD68 (to track monocyte lineages and macrophages) and IL-6. High-resolution images of the complete defect were created by the automated linking of single image frames using a Biorevo BZ-9000 microscope (Keyence, Neu-Isenburg, Germany) and the BZII Analyzer version 1.42 software (Keyence, Neu-Isenburg, Germany).

### 2.12. Detection of Transplanted Donor Cells: Y-Chromosome In Situ Hybridization

A digoxigenin (DIG)-labeled 200 bp probe to target rat Y-chromosome was synthesized utilizing DIG-high prime DNA labeling and detection starter kit I (Sigma-Aldrich, Munich, Germany) according to the protocol provided by the manufacturer. Y-chromosome in situ hybridization was performed as described by Leppik et al. (2020) [14]. Stained sections were analyzed at 100× magnification with a BZII Analyzer microscope (Keyence, Neu-Isenburg, Germany).

### 2.13. New Bone Formation Assessment

To assess the formation of new bone, 3 μm tissue sections were taken parallel to the long axis of the femora and stained with a Movat pentachrome staining kit (Morphisto, Offenbach am Main, Germany) [22]. Movat stain allows for a clear and vivid differentiation between typical bone healing tissue types: hematoma/fibrin appears in different shades of red, cartilage is deep green, fibrous connective tissue is light green-blue, and bony tissue is stained yellow [23]. Detailed images of the entire defect were generated using an automated process of linking single image frames using a Biorevo BZ-9000 microscope (Keyence, Neu-Isenburg, Germany) and BZII Analyzer version 1.42 software (Keyence, Neu-Isenburg, Germany). For each bone defect, the size of the new bone, cartilage, and fibrous tissue areas were measured by means of “color threshold” and “area” measurement options of ImageJ version 1.53g software (National Institutes of Health, Bethesda, MD, USA) [20]. These values were normalized to the size of the whole defect area. At least three slides per animal were employed for subsequent statistical analysis.

### 2.14. Immunohistochemistry

Tissue sections were deparaffinized, rehydrated, and treated to block endogenous peroxidase activity prior to antibody incubation. Tissue samples were then incubated with mouse monoclonal anti-α-SMA antibody (1 h, 2 µg/mL, 75 µL/slide, clone 1A4, Abcam, Cambridge, UK), rabbit monoclonal anti-CD68 antibody (12 h, 2 µg/mL, 75 µL/slide, clone EPR23917-164, Abcam, Cambridge, UK), mouse monoclonal anti-IL-6 (12 h, 2 µg/mL, 75 µL/slide, clone 1.2-2B11-2G10, Abcam, Cambridge, UK), or without primary antibody (negative control). As the secondary antibody, a polyclonal HRP-conjugated goat anti-mouse IgG (Histofine Simple Stain MAX PO, Nichirei, Tokyo, Japan) or a polyclonal HRP-conjugated goat anti-rabbit IgG H&L (ab6721, Abcam, Cambridge, UK) were applied for 30 min followed by incubation with 3-amino-9-ethylcarbazole (Sigma, Darmstadt, Germany) according to the manufacturer’s instructions. Finally, the tissues were counterstained with hematoxylin. Sample imaging was performed using a Biorevo BZ-9000 microscope (Keyence, Neu-Isenburg, Germany) at 40× magnification. α-SMA and IL-6-positive stained tissue areas were measured by means of “color threshold” and the “area” measurement options of ImageJ version 1.53g software (National Institutes of Health, Bethesda, MD, USA) [20]. CD68+ cells were manually counted on five standardized regions of interest (ROIs—324 μm × 240 μm) by two independent trained examiners blinded to the treatment. Mean values from every animal were calculated and used for subsequent statistical analysis.

### 2.15. In Vivo Gene Expression of Osteogenic and Inflammation Markers

The gene expression assessment was performed on defect tissues from the 1 week healing time point. Total RNA from the frozen whole content between the fracture ends was isolated using QIAzol Lysis Reagent (Qiagen, Hilden, Germany) following the instructions provided by the manufacturer. Genomic DNA was removed using RNase-free DNaseI (New England BioLabs GmbH, Frankfurt am Main, Germany) according to the manufacturer’s protocol. cDNA was synthesized from DNase-treated RNA using an iScript^®^ cDNA synthesis kit (Bio-Rad, Hercules, CA, USA). The qRT-PCR reaction was performed using the cDNA equivalent to 10 ng RNA and the iTaq Universal SYBR Green Supermix (Bio-Rad, Hercules, CA, USA). All samples were amplified in duplicates using a CFX96 Touch Real Time PCR Detection System (Bio-Rad, Hercules, CA, USA) with rat-gene-specific primers (Qiagen, Hilden, Germany), described in Supplementary Tables S1 and S2. Ribosomal protein lateral stalk subunit P1 (*Rplp1*) and tyrosine 3-monooxygenase/tryptophan 5-monooxygenase activation protein zeta (*Ywhaz*) were both used as housekeeping genes [24]. An analysis of melting curves was performed to ensure the specificity of the PCR products. The relative expression of target genes was calculated using the comparative CT (threshold cycle values) method (2^−∆Ct^) [25]. Between five and seven samples for each group were analyzed.

### 2.16. Statistical Analysis

BiAS 11.12 (Epsilon-Verlag, Darmstadt, Germany) and Graph Pad Prism 5.0 (Graph Pad Software, Inc., San Diego, CA, USA) software were employed for statistical assessment and graphic design, respectively. The results were presented either as mean values and standard deviation or as a box plot of the median (box: borders are 25% quartile and 75% quartile; whiskers represent minimum and maximum) in diagrams and as mean values and standard deviation in the text. A minimum of three replicates per group for the in vitro and six animals per group for the in vivo experiment analysis were used. The nonparametric Mann–Whitney U test was used for the comparison of two groups, and the nonparametric Kruskal–Wallis test with Bonferroni–Holm corrected post hoc analysis was employed for comparisons involving more than two groups. In all cases, a significant difference between groups was considered when the *p* value was lower than 0.05.

## 3. Results

### 3.1. Seeding Efficiency

**Figure 3:**
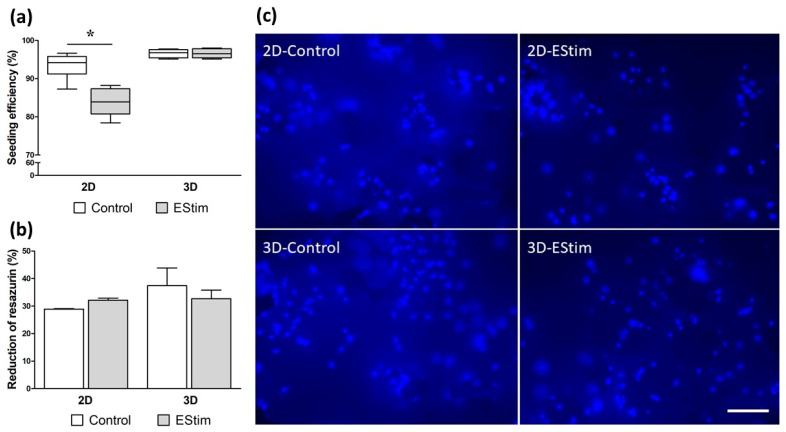
Assessment of cell seeding efficiency and metabolic activity. (**a**) Percentage of cells adhered to the scaffold material after 1 h (2D-groups, measured on experimental day 9) or 24 h (3D-groups, measured on experimental day 1) of incubation. Seeding efficiency was calculated as the difference between the number of cells initially seeded (2 × 10^5^ cells pro 90 mg β-TCP granules) and the number of cells recovered from the bottom of the plate after the incubation period and expressed as percentage. *n* = 6 replicates/group. * *p* < 0.05. (**b**) Percentage of reduction in resazurin as an indicator of cell metabolic activity in 3D constructs prior to implantation into bone defects. *n* = 3 replicates/group. (**c**) Distribution of rat male MSCs (DAPI staining) on β-TCP granules prior to implantation into critical size femur defects. Scale bar = 100 µm. EStim, electrical stimulation group.

The percentage of cells adhered to the scaffold material after 24 h (3D-group, experimental day 1) or 1 h (2D-groups, experimental day 9) of incubation is shown in Figure 3a. All groups showed percentages higher than 93% except the 2D-EStim group, where the pretreatment with EStim for 1 week significantly reduced this parameter (*p* < 0.05).

### 3.2. Metabolic Activity

The percentage of reduction in resazurin (alamarBlue^®^) was measured as an indicator of cell metabolic activity on 3D constructs prior to their transfer into bone defects (Figure 3b). The pretreatment of MSCs with EStim for 1 week in 2D and 3D culture conditions did not modify this parameter (*p* > 0.05). Furthermore, no differences were detected when comparing the values from the 2D and 3D culture conditions (*p* > 0.05).

### 3.3. Cell Distribution on 3D Constructs

DAPI staining of cell nuclei before implanting the constructs into bone defects revealed the adherence and uniform distribution of cells on the β-TCP scaffold granules among the different treatment groups (Figure 3c).

### 3.4. Donor Cell Detection in Defect Tissues

To identify donor cells transplanted from male rats, the defect tissues were hybridized with a DIG-labeled probe specific for Y-chromosome SRY1 gene. The qualitative analysis performed on the tissue sections from the defect area showed positively stained donor cells in all groups and healing time points (Supplementary Figure S1).

### 3.5. µCT Analysis

**Figure 4:**
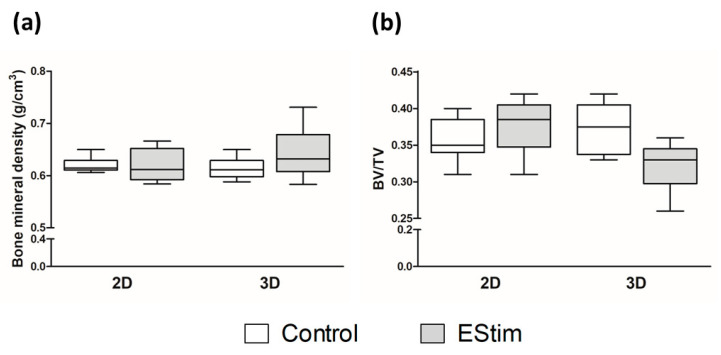
Radiological analysis of bone defects. (**a**) Bone mineral density of bone defects at 8 weeks post-surgery. (**b**) Ratio of new bone volume (BV) to defect total volume (TV), as quantified at 8 weeks post-surgery. EStim, electrical stimulation groups. *N* = 9–16 animals per group.

**Figure 5:**
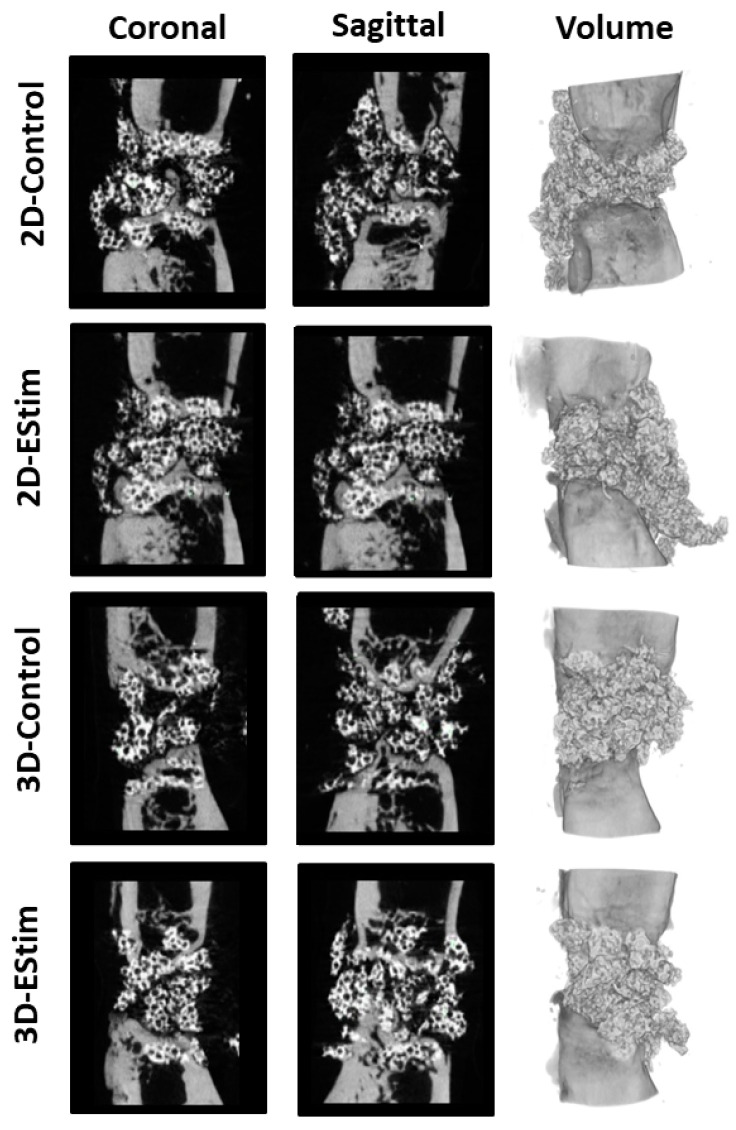
Representative sectional images and 3D reconstructions of µCT scans of femora collected at the 8-week healing time point. Left column: coronal sections; middle column: sagittal sections; and right column: 3D reconstructions (volume). Similar to histological analysis, sectional images show comparable bone formation in the different groups, but complete bone bridging has not yet occurred. In the volume reconstructions, new bone formation and the radiopaque β-TCP granules can be observed.

The amount of new bone in the defect was assessed at the 8-week healing time point by means of µCT analysis and the bone mineral density (Figure 4a) and the ratio of new bone volume to total defect volume were calculated (Figure 4b). Although the values of bone mineral density were slightly higher in the 3D-EStim group in comparison to the other groups, they did not significantly differ. Furthermore, comparisons of the ratio of new bone volume/total defect volume from the different groups showed no significant differences. Representative sectional images and 3D reconstructions are shown in Figure 5. Similar to histological analysis, sectional images show similar bone formation in the different groups, but full bridging of the bone defect had not yet taken place. New bone formation and β-TCP granules can also be observed in the volume reconstruction.

### 3.6. Bone Mechanical Strength

**Figure 6:**
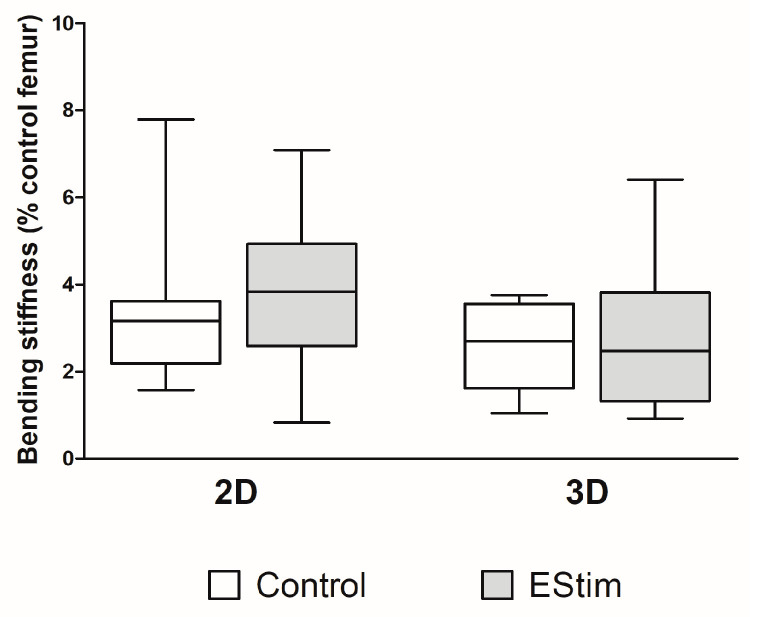
Mechanical analysis of bone defects. Biomechanical strength (bending stiffness) of the defect areas measured by destructive 3-point bending test at the 8-week healing time point. EStim, electrical stimulation groups. *N* = 9–16 animals per group.

The mechanical properties of newly formed bone at the 8-week healing time point were assessed using a destructive three-point bending test, and the results are shown in Figure 6. Although the median value from the 2D-EStim group was higher in comparison to the median values from the other groups, they did not significantly differ.

### 3.7. Histological and Immunohistological Assessment of Bone Healing

**Figure 7:**
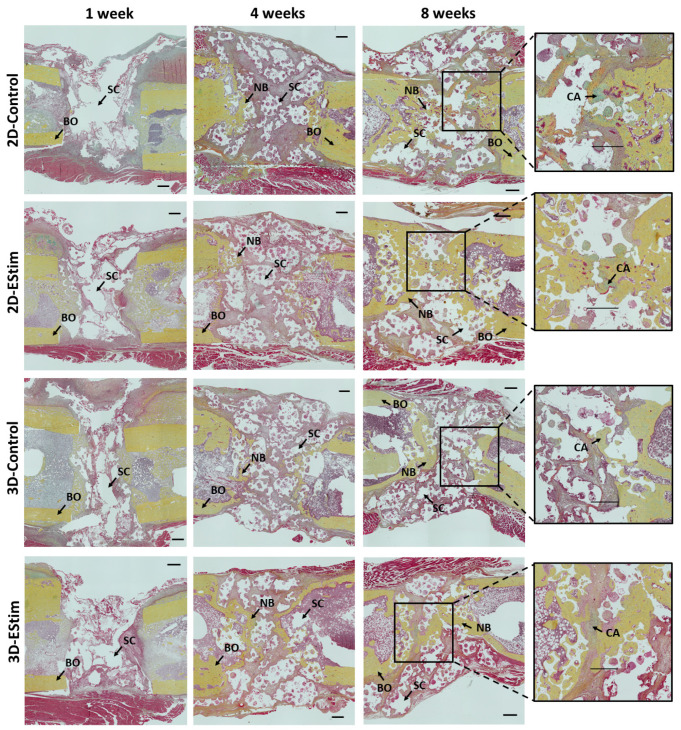
Representative histological images of femur defects collected at the 1-, 4-, and 8-week healing time points. Histological cuts were stained with Movat Pentachrome. EStim, electrical stimulation groups. BO = bone; NB = newly formed bone tissue; SC = β-TCP scaffold; CA = cartilage. Scale bar = 500 μm.

The histological analysis of bone defects was performed at 1, 4, and 8 weeks post-surgery (Figure 7). At the 1-week healing time point, bone defects in all groups were mainly filled by undifferentiated fibrous tissue (granulation tissue), while at the 4- and 8-week healing time points, an increasing amount of new bone tissue was visible in the bone defect area.

**Figure 8:**
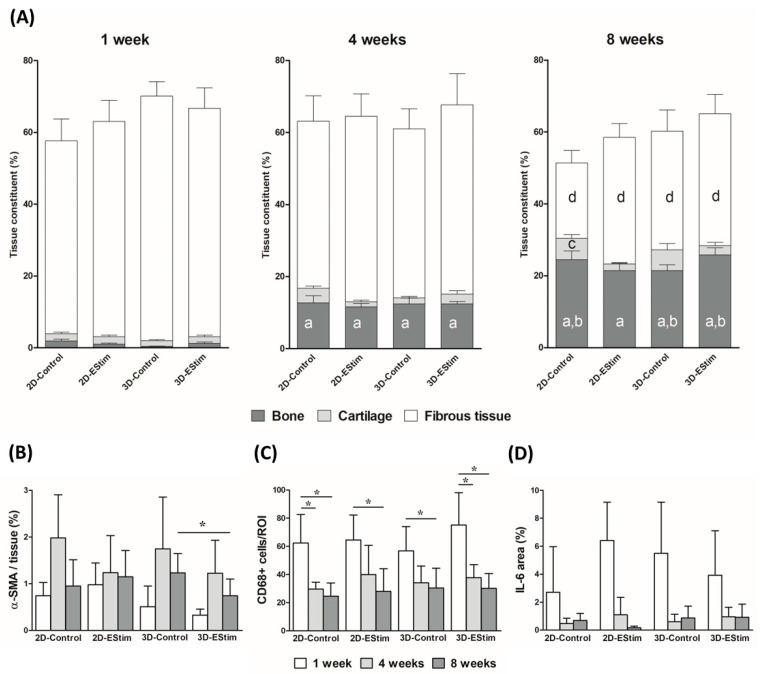
Histomorphometric and immunohistological analysis of bone defects. (**A**) Tissue composition within the defect area at the 1-, 4-, and 8-week healing time points. (**B**) Vessel density, (**C**) CD68+ cells, and (**D**) IL-6-positive area in defect tissues at 1, 4, and 8 weeks post-surgery. Data are expressed as mean ± SD. *n* = 9–16 animals per group. a: *p* < 0.05 vs. bone tissue from the same group at 1 week. b: *p* < 0.05 vs. bone tissue from the same group at 4 weeks. c: *p* < 0.05 vs. cartilaginous tissue from the same group at 1 week. d: *p* < 0.05 vs. fibrous tissue from the same group at 1 week. *: *p* < 0.05.

The proportion of new bone, cartilage, and fibrous tissue in the defect area at the three healing time points is shown in Figure 8A. The percentage of new bone tissue increased across the healing time points (1 vs. 4 vs. 8 weeks; *p* < 0.05), and at 1 and 8 weeks, the mean values were higher in the 3D-EStim compared to the 3D-Control group. Nevertheless, these differences did not reach statistical significance. The percentage of cartilage was significantly higher in the 2D-Control group at 8 weeks vs. 1 week (*p* < 0.05), while no significant differences were visible when comparing the healing periods from the other groups. The percentage of fibrous tissue was lower at 8 weeks in all groups compared to the same respective group at 1 week (*p* < 0.05), but the differences between EStim and the control groups at the same healing time points were not significant.

To assess the effect of EStim on new vessel formation, histological sections of the defects from the 1-, 4-, and 8-week healing time points were stained with anti-α-SMA antibody. The vessel density was measured and compared to the total tissue area in the defects (Figure 8B and Supplementary Figure S2). At the 8-week healing time point, the 3D-EStim group showed lower vessel density vs. the 3D-Control group (*p* < 0.05). Comparisons between groups at the 1- or 4-week time points as well as comparisons between the three time points from every group showed no significant differences.

The inflammatory pattern among the healing time points and groups was assessed through immunohistochemistry for CD68 and IL-6. The count of CD68+ cells (monocyte lineages and macrophages) per region of interest in the tissue sections of bone defects (Figure 8C and Supplementary Figure S3) decreased over time (1 vs. 4 vs. 8 weeks; *p* < 0.05). Nevertheless, there were no differences produced by the pretreatment of MSCs with EStim. The IL-6-positive area is shown in Figure 8D and Supplementary Figure S4. This variable tended to be higher in 2D-EStim vs. 2D-Control at 1 week (*p* = 0.08) while all the other statistical comparisons (between groups and time points) showed no significant differences.

### 3.8. In Vivo Gene Expression of Osteogenic and Inflammation Markers

**Figure 9:**
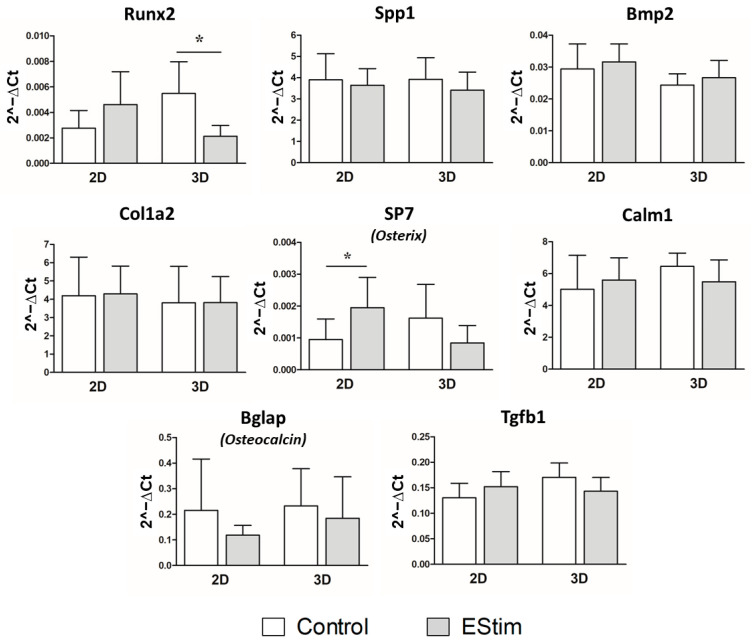
Gene expression of osteogenic markers in rat bone defects 1 week after implantation of constructs. Gene expression of *RunX2*, *Spp1*, *Bmp2*, *Col1a2*, *SP7* (*Osterix*), *Calm1*, *Bglap* (*Osteocalcin*), and *Tgfb1* was quantified by means of RT-qPCR. Data are expressed as mean ± SD. *N* = 5–7 animals per group. * *p* < 0.05.

**Figure 10:**
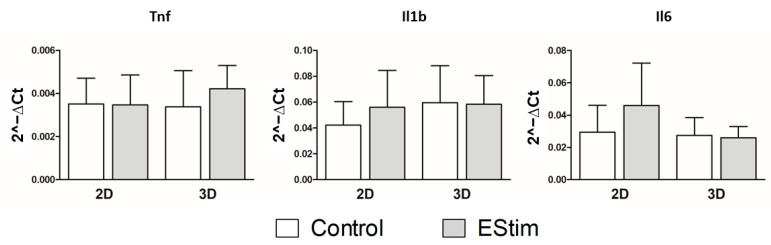
Gene expression of inflammation markers in rat bone defects 1 week after implantation of constructs. Gene expression of *Tnf*, *Il1b*, and *Il6* was quantified by means of RT-qPCRs. Data are expressed as mean ± SD. *N* = 5–7 animals per group.

Gene expression of osteogenic and inflammation markers was assessed by qPCR on bone defects after 1 week of healing and are shown in Figure 9 and Figure 10, respectively. At this time point, the expression of *Runx2* gene was downregulated in the 3D-group pretreated with EStim for 1 week (*p* < 0.05) while the gene expression of *Osterix* was upregulated in the 2D-EStim group (*p* < 0.05). The other markers of osteogenic differentiation (*Spp1*, *Bmp2*, *Osteocalcin*, *Col1a2*, *Osterix*, *Tgfb1*, *Calm1*) and inflammation (*Tnf*, *IL-1b*, and *IL-6*) did not significantly differ when comparing the control and experimental groups or culture conditions 1 week after fracture.

## 4. Discussion

In the present study, we assessed whether the pretreatment of MSCs with EStim in 2D or 3D culture conditions improved the healing of a 5 mm segmental bone defect in rat femora. We found that pretreating MSCs with EStim in both 2D and 3D scenarios did not modify healing outcomes, as only slight improvements were observed over an 8-week observation period, compared to non-stimulated control MSCs. As expected, all the groups experienced a considerable increase in the proportion of new bone tissue in the defects over the three healing time points studied, with a concurrent decline in the percentage of fibrotic tissue and IL-6. These findings are in line with our earlier observations made in a rat critical size femur defect model [16,21].

There are several possible explanations for the differences we observed between the positive pro-osteogenic findings in our previous in vitro experiments and the present in vivo study. The most obvious reason is the difference between the local environment in the in vitro cell culture setting, and the tissue-specific environment within the in vivo healing bone defect, which could have counteracted the pro-osteogenic effect of EStim on MSCs. Furthermore, the previously observed positive influence of EStim in vivo could result from its effect on the entire healing environment, which cannot be mimicked by treating only one part or player of this environment (MSCs).

Prior to implanting the MSCs + bone graft substitute constructs into the defects, we assessed the seeding efficiency and cell metabolic activity. Although the seeding efficiency was lower in the 2D-EStim group, the metabolic activity remained unchanged or even showed a slight increase compared to its respective control. It has been suggested that EStim can increase cell metabolic activity, leading to the intracellular depletion of ATP. This, in turn, can alter the function of linker proteins responsible for the physical attachment between the membrane and cytoskeleton [26]. Additionally, other studies have described a positive effect of EStim on cell adhesion [27]; however, this effect was seen only in response to a significant electrical stimulus [28]. In both of these cases, cells were stimulated after being seeded onto the material, unlike in our 2D-EStim group, where cells were stimulated before being seeded onto scaffold granules.

Oxygen concentrations in in vitro versus in vivo settings may differ and effect the MSCs osteogenic activity. In the present study, as well as in our previous studies [12], we treated cells with EStim at an oxygen concentration of 5%, which mimics physiological oxygen values of niches where MSCs reside in vivo [29]. It has been described that comparable hypoxic conditions can lead to the increased expression of osteogenic markers in MSCs [30,31]. Leach’s group reported that hypoxic preconditioned MSCs, when implanted in an alginate hydrogel into femoral defects in athymic rats, improved bone healing. In said study, they preconditioned their MSCs at 1% oxygen for 3 days [32]. In the present, and in our previous studies [12], the available oxygen for MSCs cultured on scaffolds was most likely lower than 5%, especially in the pores and undersides of the scaffold granules, where perfusion is decreased. Although in this study we did not measure the local oxygen concentration, previous cytocompatibility analyses of various bone substitutes under similar culture conditions, over a 14-day period, showed that MSCs can survive under these conditions [33,34,35]. However, the possibility that local O_2_ partial pressure is lower in vitro, and that this can lead to altered cell behavior when transplanted into bone defects in vivo, cannot be excluded.

The presence of electrolytic faradic products in in vitro versus in vivo conditions vary and can affect osteogenic activity. In this study, we did not measure the presence of electrolytic faradic products, although we expect that they were present in comparable concentrations in our 2D and 3D cultures as both were stimulated using similar electrical parameters under comparable culture conditions. Furthermore, it has been described that low concentrations of faradic products, such as H_2_O_2_, have a supportive effect on cell function and differentiation [36,37,38,39].

Other reasons why the pretreatment with EStim did not induce pro-osteogenic activity in the MSCs in our study could be related to the immunologically active environment in the freshly created bone defect. Fresh fracture hematomas have been described to contain various activated immune cell populations, including neutrophils, monocytes/macrophages, T cells, and NK cells. Neutrophils secrete cytokines such as IL-1β, IL-6, and TNF-α (whose transcription was assessed in the present study), as well as IL-10, MCP-1, CXCL1α, and MIP-1, which attract monocytes and are involved in their differentiation into macrophages [40]. Other growth factors present in the fracture hematoma include PDGF, TGF-β, FGF-2, EGF, VEGF, IGF-1, BMP-2, -4, -6, and PF4, as well as inflammatory factors like serotonin and histamine, coagulation factors, and antimicrobial factors [41].

Macrophages play an important role in orchestrating the different phases of fracture healing, as summarized by Schlundt et al. (2021) [42]. In the present study, all groups exhibited a significant decline in macrophage density in the defect area over time, maintaining similar levels at the 4- and 8-week healing time points. We previously described the same pattern of macrophage kinetics after 8 weeks of healing in a rat femoral segmental defect model [15,43,44], as did Schlundt et al. (2018) [45] using a murine femur fracture model. In the present study, no relevant differences in the macrophage count attributable to EStim treatment were observed. This is in accordance with previous results from an in vivo study performed by our group, in which EStim did not modify the total number of macrophages but instead influenced the proportion of M1 and M2 polarized cells [44].

Gene expression analysis of the defect tissue at the 1-week postoperative time point revealed clear evidence of a pro-inflammatory milieu. In all groups, mRNA for IL-1β, TNF-α, and IL-6 could be detected. The role of various inflammatory mediators in osteogenic differentiation of MSCs is controversial. It has been described that mediators such as IL-1β and TNF-α can exert an adverse effect on the differentiation of murine MSCs into osteoblasts [46], although both mediators at low concentrations can also promote osteogenic differentiation in vitro [47,48]. Additionally, gene expression of the pleiotropic growth factor TGF-β1 was detected in all groups, and this can exert anti-inflammatory and pro- or anti-osteogenic effects depending on the context [49].

Interleukin-6 is another prominent cytokine present in higher concentrations in fracture hematoma during the early phase of bone healing, which also plays an ambivalent role in the osteogenic differentiation of MSCs. In the present work, IL-6 was studied at both the gene expression and immunohistological level over the course of 8 weeks. One week after surgery, comparable gene expression levels of IL-6 were detected in all groups. Furthermore, in all groups, a similar immunohistological pattern was observed: initial high levels, which decreased to basal levels after 4 weeks and remained constant until 8 weeks. The effect of IL-6 on MSCs has been described as heterogeneous. On one hand, IL-6 is reported to increase the ’stemness’ of MSCs, thus counteracting differentiation [50]; on the other hand, it was evidenced that MSCs express the IL-6 receptor during osteogenic differentiation. IL-6 binding to its receptor could lead to the activation of the STAT3 pathway, which facilitates the osteogenic differentiation of MSCs [51]. The latter may be consistent with the slight increase in IL-6 revealed by gene expression and immunohistological analysis in our 2D-EStim group compared to its control at the 1-week healing time point, which was associated with the up-regulation of *Osterix* and slightly better radiological and mechanical outcomes in this group.

Gene expression analyses of the defect area also indicated a pro-osteogenic environment (*Bmp2*, *Osteocalcin*, *Col1a2*, *Tgfb1*) and signs of osteogenic differentiation activity (*RunX2*, *Osterix*). Bone morphogenetic protein 2 (*Bmp2*) has been described as a highly potent mediator of osteogenic differentiation in vitro and in vivo [52]. Transforming growth factor beta 1 (*Tgfb1*) is important for the induction of osteogenesis but may act as an inhibitor at later stages of differentiation [46,53]. Collagen type I is the major protein component of the extracellular bone matrix and accounts for up to 90% of the organic matrix [54]. The matrix protein osteocalcin is primarily produced by osteoblasts and regulates the mineralization of bone matrix [55].

In the present study, the expression of osteogenic gene markers did not differ between the control and treatment groups, except for the abovementioned expression of the osteogenic transcription factor *Osterix*, which was increased in the 2D-EStim group compared to its respective control. Additionally, *RunX2* gene expression was significantly lower in the 3D-EStim group than in its corresponding control. However, the contribution of the implanted MSCs to gene expression patterns remains unclear. No conclusion can be drawn as to whether the observed changes can be primarily attributed to the implanted cells or whether they are due to secondary effects mediated by the inserted cells. The observed increase in gene activity of the transcription factor *Osterix* in the 2D-EStim group may be related to the slightly higher percentage of new bone volume and bending stiffness observed in this group, while the decreased gene activity of the transcription factor *RunX2* in the 3D-EStim group could be related to the slightly lower percentage of new bone volume radiologically observed in this group. Both transcription factors are essential for the formation of new bone tissue, as indicated in this review article [56].

When considering the primary outcome “bending stiffness”, very similar results were found in all groups after the 8-week healing period: neither EStim nor the 3D culture before construct implantation produced significant variations. However, as mentioned before, in the group whose cells were pretreated with EStim in 2D culture, slightly increased values in terms of bending stiffness and the percentage of new bone in the total defect volume (measured by µCT) were observed. 

To our knowledge, the effects of a complex combination of different mediators on the maintenance of a previously induced pro-osteogenic differentiation potential have not yet been investigated. It can be speculated that the interaction of several players may inhibit or reverse the osteogenic differentiation of MSCs, or even possibly induce transdifferentiation to another cell type like endothelial cells through mediators like VEGF [57]. 

Another important aspect in large bone defect healing is the rapid establishment of a vascular network to supply the defect zone, as well as the subsequent reduction in the vascular density, later in the further course of healing [58]. In this regard, we observed lower vascularization in the 3D-EStim group at the 8-week healing time point compared to its control group. Nevertheless, this change was not accompanied by lower fibrous tissue in the histological analysis, which, in turn, could have been advantageous for the bone healing outcomes of this group. When considering the other time points and experimental groups, no relevant differences were detected. A possible explanation is that MSCs in general do not significantly support vascularization in this animal model, as it was observed in one of our previous studies at the 1- and 8-week postoperative time points [59].

In the present study, we hypothesized that by pretreating MSCs with EStim, we would improve BTE outcomes by harnessing the positive effects of EStim on bone healing, while obviating the need for a surgically implanted EStim device, and thus simplifying the procedures at the bed side level, reducing the risk of complications and the associated high costs. When considering alternatives to this strategy, the use of electroactive scaffolds emerges as one that is worth exploring further. Recently, new BTE treatment approaches incorporating conductive materials have been proposed. These materials have the capacity to transfer electrical and electromechanical signals to cells (reviewed in [60]). Several in vitro and in vivo studies have already shown improvements in cellular behavior and bone formation using these methods (reviewed in [61]). Although more advances in the field are still needed to demonstrate the long-term capabilities of the electroactive scaffolds (reviewed in [62]), these treatments represent promising alternatives for the therapeutic management of delayed and non-healing large bone defects and could lead to the widespread application of this technology.

## 5. Conclusions

The strategy of pretreating MSCs with EStim to improve BTE treatments seemed promising but failed to be effective in our in vivo rat femur defect model. The long-term, pro-osteogenic effects we observed in vitro, using EStim, could not be reproduced in vivo. Although negative, these results are of great importance for the ongoing development of new cell-assisted EStim and BTE therapies. These findings should be taken into account when considering EStim treatments for promoting regenerative processes. As demonstrated in our previous in vivo studies using a rat femur defect model, to effectively improve bone healing, EStim needs to be administered directly and continuously at the defect site throughout the healing process.

## Data Availability

Not applicable.

## Supplementary Materials

The following supporting information can be downloaded at: https://www.mdpi.com/article/10.3390/cells12172151/s1, Figure S1: Detection of transplanted donor cells in tissue sections of bone defects at the 1-, 4-, and 8-week healing time points; Figure S2: Representative images of α-smooth muscle actin (α-SMA) immunohistochemical staining in femur defect tissues collected at the 1-, 4-, and 8-week healing time points; Figure S3: Representative images of CD68 immunohistochemical staining in femur defect tissues collected at the 1-, 4-, and 8-week healing time points; Figure S4: Representative images of IL-6-positive area in femur defect tissues at 1, 4, and 8 weeks post-surgery; Table S1: Description of commercially available primers; and Table S2: RT-qPCR primer sequences.
