# Supplementary material for: Pretreatment of Mesenchymal Stem Cells with Electrical Stimulation as a Strategy to Improve Bone Tissue Engineering Outcomes
Source: Cells. 2023 Aug 26;12(17):2151. doi: 10.3390/cells12172151 (PMC10487010; doi:10.3390/cells12172151)
Supplement: Supplementary file 1 [file cells-12-02151-s001.zip › cells-2542854-supplementary.pdf]

Supplementary information

Pretreatment of Mesenchymal Stem Cells with Electrical Stimulation as a Strategy to Improve Bone Tissue Engineering Outcomes.

Santiago Bianconi, Karla M. C. Oliveira, Kari-Leticia Klein, Jakob Wolf, Alexander Schaible, Katrin Schröder, John Barker, Ingo Marzi, Liudmila Leppik, Dirk Henrich.

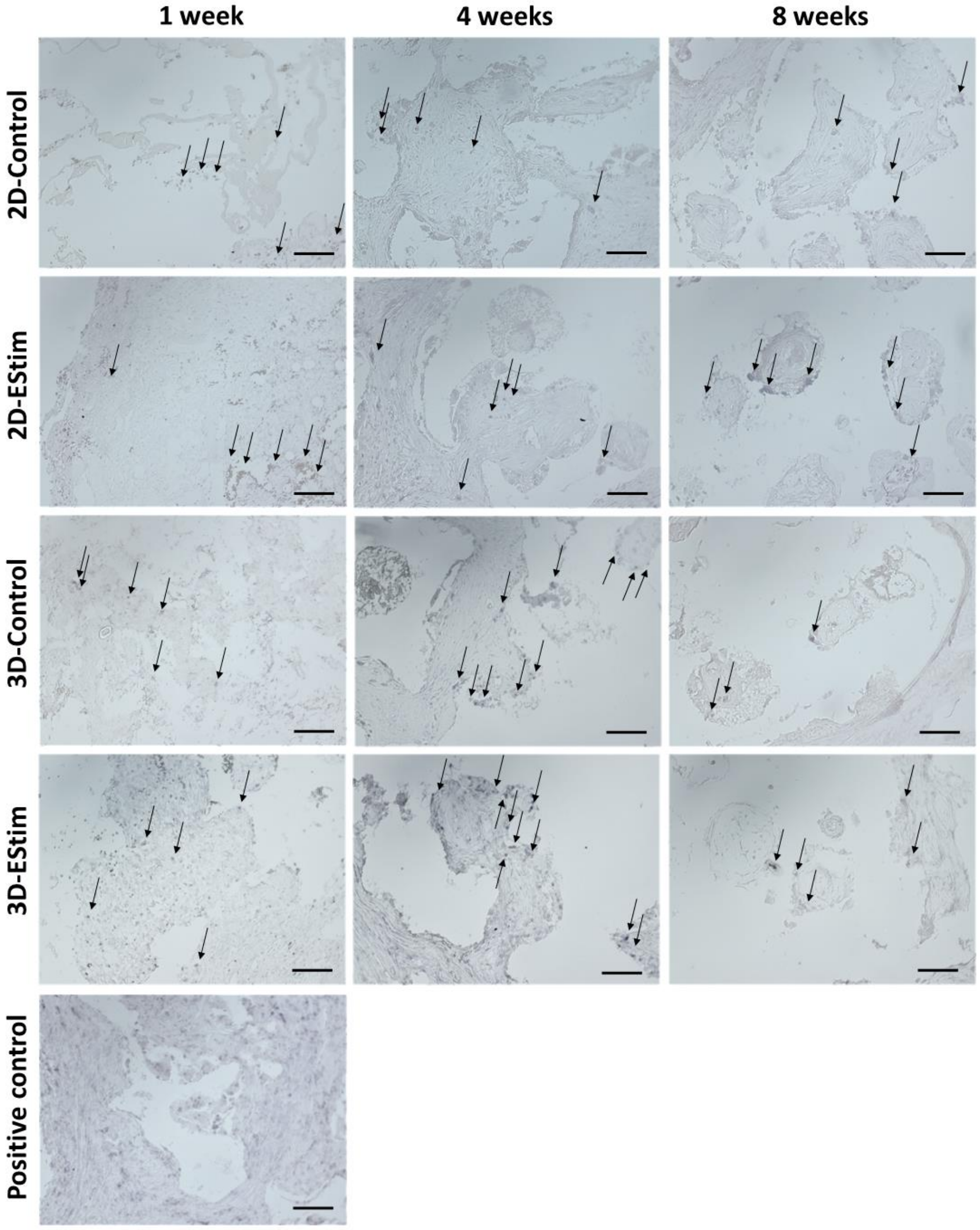

Supplementary Figure S1. Detection of transplanted donor cells in tissue sections of bone defects at the 1-, 4- and 8-week healing time points. Histological sections were hybridized with male-specific digoxigenin-labeled SRY1-probe and counterstained with nuclear fast red solution. As positive control, a histological section of male bone defect was used. Black arrows indicate the presence of male cells (dark violet nuclei). EStim, electrical stimulation groups. Scale bar = 100 μm.

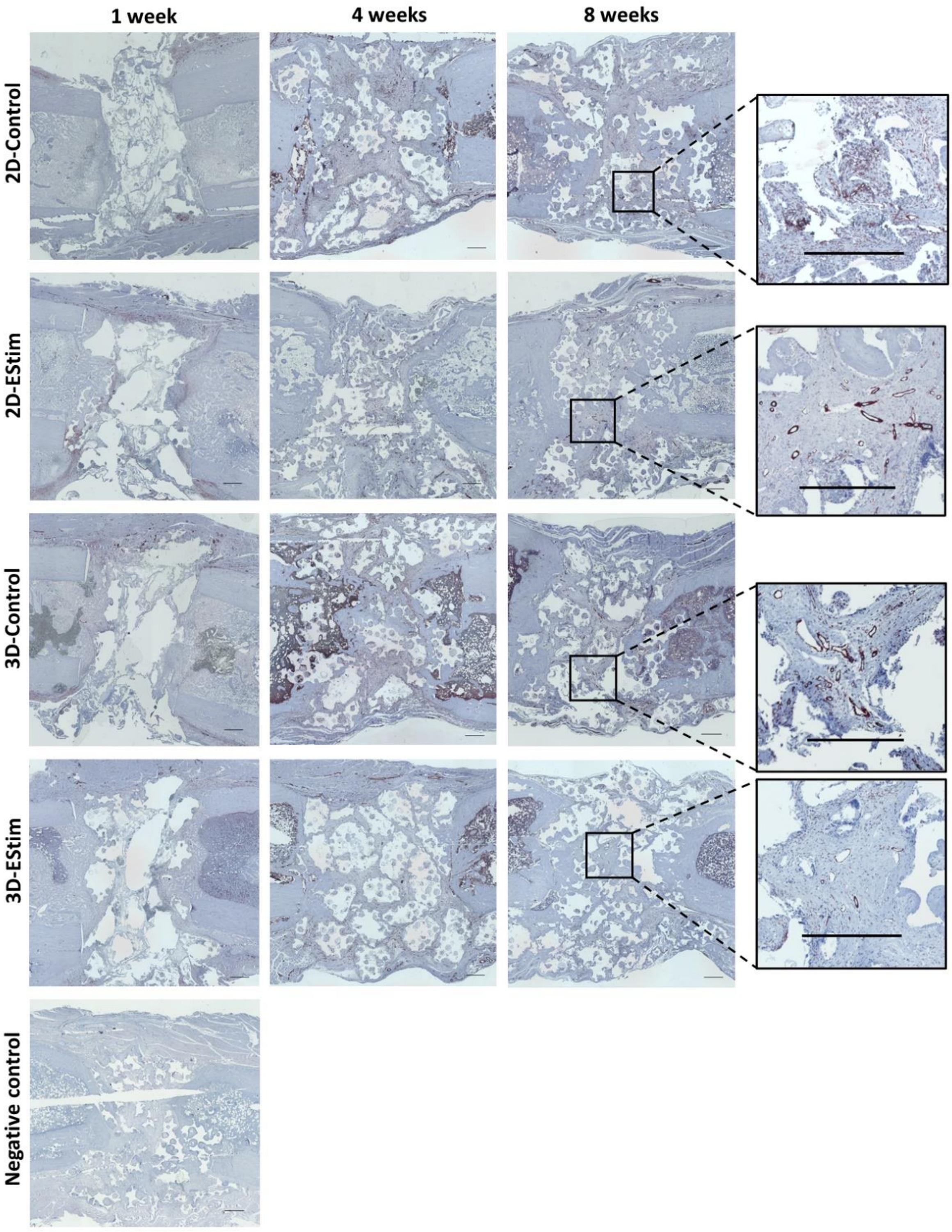

Supplementary Figure S2. Representative images of α-smooth muscle actin (α-SMA) immunohistochemical staining in femur defect tissues collected at the 1-, 4- and 8-week healing time points. Histological sections were stained with antibody anti-α-SMA and positive stained tissue areas were measured using "color threshold" and "area" measurement options in ImageJ software. EStim, electrical stimulation groups. Scale bar = 500 µm.

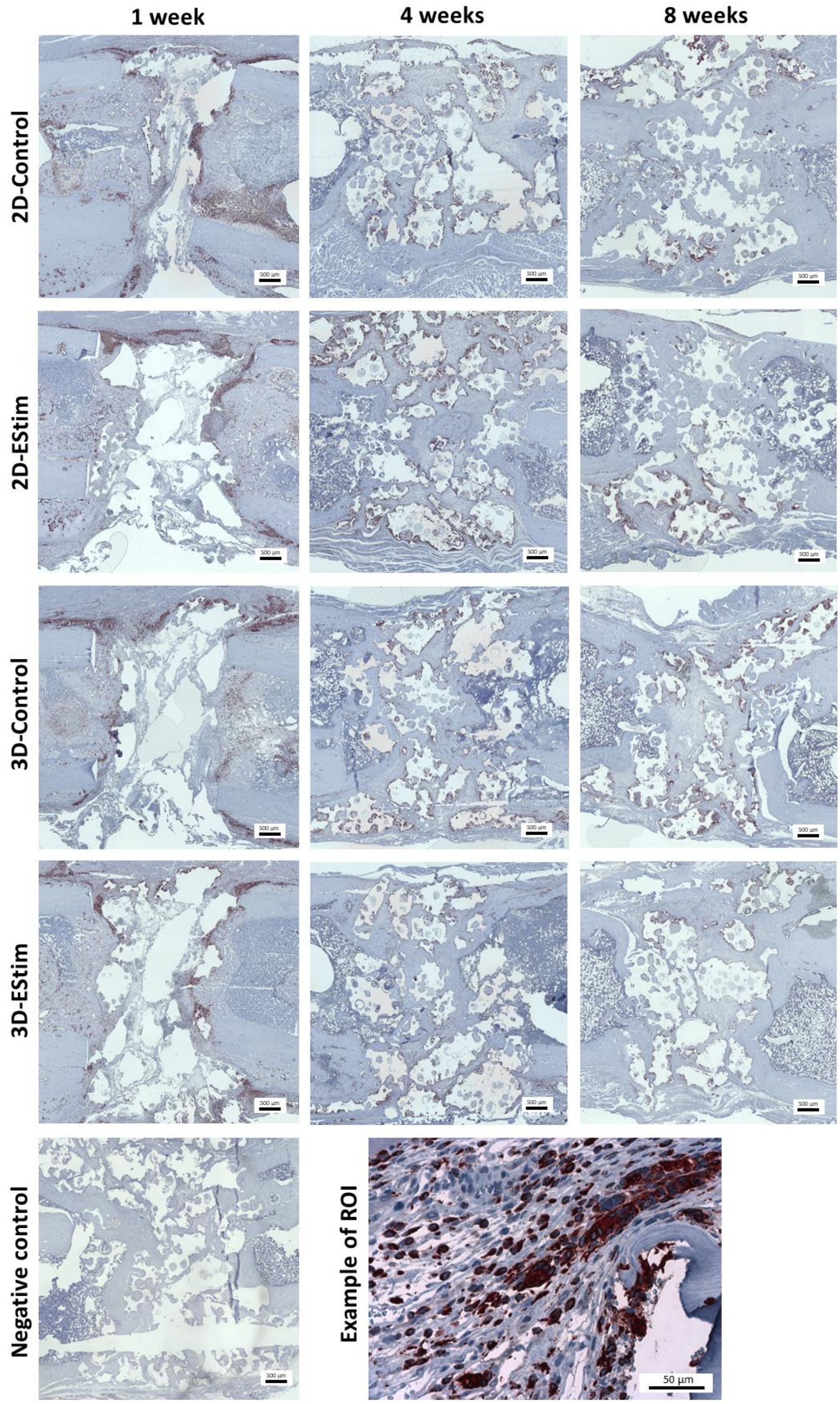

Supplementary Figure S3. Representative images of CD68 immunohistochemical staining in femur defect tissues collected at the 1-, 4- and 8-week healing time points. Histological sections were stained with antibody anti-CD68 and CD68+ cells (monocyte lineages and macrophages) were counted manually in five regions of interest (ROI) per sample, at 200x magnification. EStim, electrical stimulation groups.

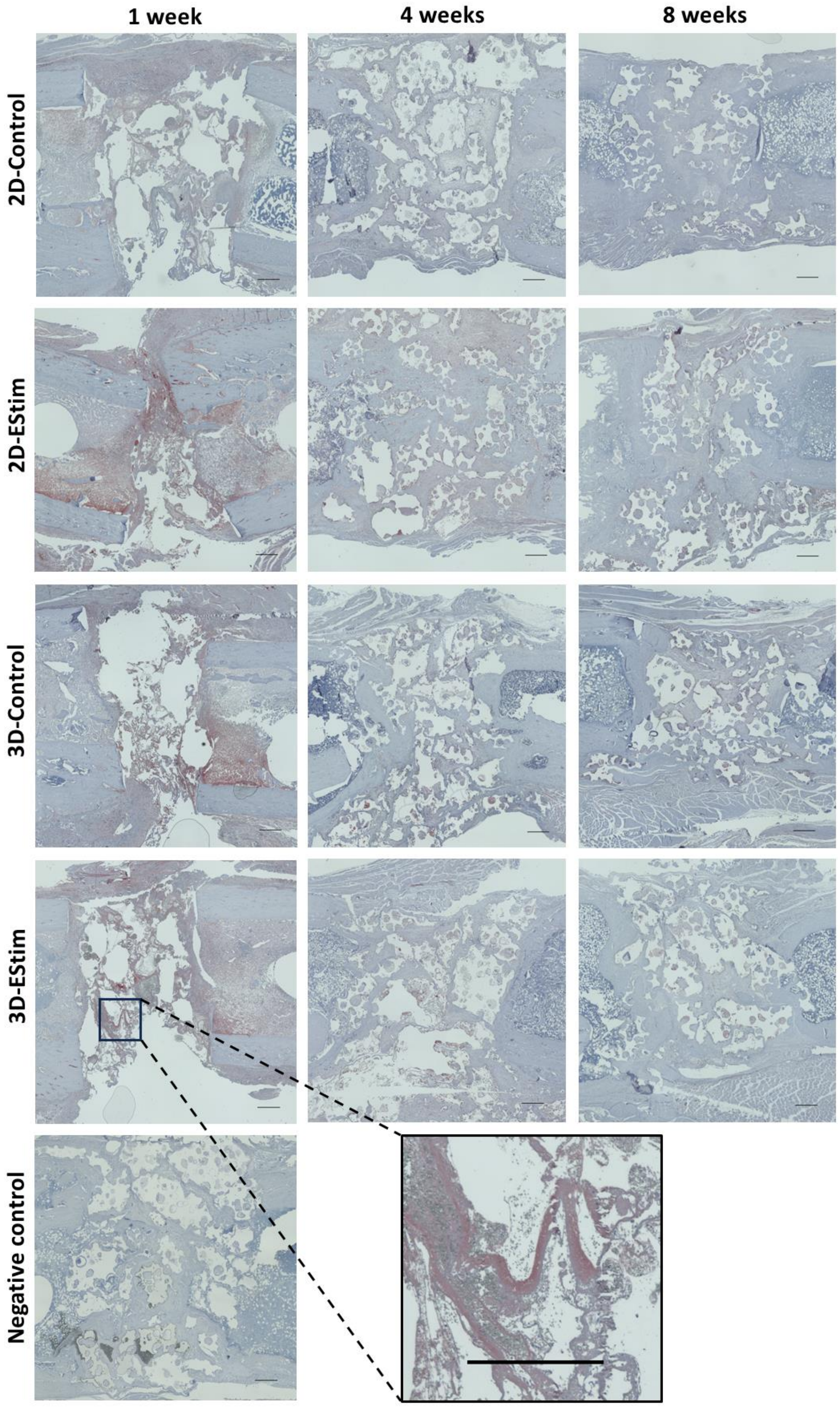

Supplementary Figure S4. Representative images of IL-6 positive area in femur defect tissues at 1-, 4- and 8-weeks post-surgery. Histological cuts were stained with antibody anti-IL-6 and positive stained tissue areas were measured using "color threshold" and "area" measurement options in ImageJ software. Scale bar = 500 μm.

Supplementary table S1. Description of commercially available primers

GeneGlobe Id:	Abbreviation	Name
PPR06531B-200	Bmp2	Bone morphogenetic protein 2
PPR06480B-200	Il1b	Interleukin 1 beta
PPR06483B-200	Il6	Interleukin 6
PPR53039B-200	Runx2	RUNX family transcription factor 2
PPR06411F-200	Tnf	Tumor necrosis factor

Supplementary table S2. RT-qPCR primer sequences

Gene	Forward (5′ → 3′)	Reverse (5′ → 3′)	Annealing temperature (◦C)
Calm1	TTTGACAAGGATGGCAATGGCT	TGTTAGCTTTTCCCCGAGGT	56
Col1a2	TTCCCGGTGAATTCGGTCT	ACCTCGGATTCCAATAGGACCAG	62
Bglap	GGAGGGCAGTAAGGTGGTGA	GAAGCCAATGTGGTCCGC	62
SP7	CTGGGAAAAGGAGGCACAAAG	GGGTGGGTAGTCATTGGCATAG	62
Spp1	GATGAACAGTATCCCGATGCC	TCCAGCTGACTTGACTCATGG	62
Tgfb1	CTGCTGACCCCCACTGATAC	AGCCCTGTATTCCGTCTCCT	59
Rplp1	GCATCTACTCCGCCCTCATC	GCATCTACTCCGCCCTCATC	64
Ywhaz	GATGAAGCCATTGCTGAACTTG	GTCTCCTTGGGTATCCGATGTC	66

Calm1, calmodulin 1; Col1a2, collagen type I alpha 2 chain; Bglap, osteocalcin; SP7, SP7 transcription factor (osterix); Spp1, secreted phosphoprotein 1 (osteopontin); Tgfb1, transforming growth factor, beta 1; Rplp1, Ribosomal protein P1; Ywhaz, Tyrosine 3-monooxygenase/tryptophan 5-monooxygenase activation protein zeta.
